# Evaluation of Analgesia Using Perineural Dexamethasone Compound in Interscalene Brachial Plexus Block After Shoulder Surgery

**DOI:** 10.5812/aapm-142635

**Published:** 2024-02-15

**Authors:** Mahshid Ghasemi, Arman Janparvar, Faranak Behnaz, Farinaz Taheri

**Affiliations:** 1 Anesthesiology Research Center, Shahid Beheshti University of Medical Sciences, Tehran, Iran; 2 Shohada Tajrish Hospital, Shahid Beheshti University of Medical Sciences, Tehran, Iran

**Keywords:** Shoulder Surgery, Non-opioid Medication, Anesthesia, Side Effects

## Abstract

**Background:**

The objective of this study was to examine analgesia when using perineural dexamethasone compound in an interscalene brachial plexus block following shoulder surgery.

**Methods:**

This study was designed as a randomized, double-blind clinical trial. Patients meeting the specified criteria were randomly divided into two groups: The experimental group and the control group, each comprising 30 individuals. Age and gender were matched between the groups. The control group received lidocaine along with 2 cc of 0.5% bupivacaine (20 milligrams) and 2 cc of normal saline; however, the experimental group received lidocaine, along with 2 cc of 0.5% bupivacaine and 2 cc of dexamethasone. Pain levels were assessed using the Visual Analog Scale (VAS), and covariance analysis was applied for data analysis.

**Results:**

The results demonstrated that pain intensity was notably lower in the experimental (dexamethasone) group than in the control group at both the 12-hour group (P < 0.001) and 24-hour (P < 0.001) postoperative marks. Dexamethasone significantly reduced pain among the patients.

**Conclusions:**

In conclusion, administering dexamethasone to potential candidates for shoulder surgery could lead to prolonged analgesia for up to 24 hours after the surgery. Consequently, this medication can serve as an efficacious analgesic option for pain management in these patients.

## 1. Background

Insufficient postoperative pain management remains a significant challenge following various surgeries, leading to adverse outcomes, such as chronic postoperative pain. Optimal postoperative pain management necessitates a comprehensive understanding of pain pathophysiology, the invasive nature of surgical procedures, and patient-related factors contributing to increased pain, such as anxiety and depression (1). Some studies have shown that intractable pain has negative effects, including coronary artery ischemia, hyperactivity of the sympathetic adrenal system, deep vein thrombosis, inadequate breathing depth, atelectasis, increased heart rate, and elevated blood pressure. Given the side effects of opioid agents in postoperative pain control, specialists are seeking non-opioid drugs that have fewer negative effects and are more cost-effective (2, 3). Additionally, pain control and treatment after shoulder surgery pose challenges for both anesthesiologists and orthopedic surgeons. The brachial plexus block can be employed as an adjunct to general anesthesia or as a primary anesthesia method (4).

Several drugs have been studied as adjuncts to regional anesthesia, such as epinephrine, clonidine, opioid agents, and ketamine. Their effects on anesthesia and analgesia vary based on the drug used and the chosen administration site; however, the results are conflicting (5). Due to the limited efficacy or suspected toxicity of previously studied drugs, some researchers have evaluated glucocorticoids as adjuvants for regional anesthesia. Glucocorticoids possess recognized anti-inflammatory, analgesic, immunosuppressive, and antiemetic properties. Research suggests that a single dose of glucocorticoid around the time of surgery is safe (6). In certain studies, dexamethasone has been observed to potentially prolong sensory block duration and effectively reduce postoperative pain intensity, especially in opioid-consuming individuals, when used intravenously and perineurally as an adjuvant in peripheral nerve blocks for upper limb surgery (7). The question of whether dexamethasone prolongs regional anesthesia remains a topic of debate. 

Steroids induce a certain degree of vasoconstriction, similar to epinephrine, which could decrease the absorption of local anesthetics. Another hypothesis suggests that dexamethasone might act locally on type C nerve fibers, leading to increased activity to suppress potassium channels, thereby reducing their activity (4). Despite various investigations conducted to date, no study has specifically explored the analgesic effects of using the perineural dexamethasone compound in interscalene brachial plexus blocks following shoulder surgery. 

## 2. Objectives

The current study aimed to address the question of whether the perineural dexamethasone compound effectively reduces pain intensity in interscalene brachial plexus blocks after shoulder surgery.

## 3. Methods

This study was designed as a randomized, double-blind clinical trial. The study population consisted of patients classified as the American Society of Anesthesiologists (ASA) class I and II, aged between 35 and 65 years, who were candidates for shoulder surgery and referred to Akhtar and Shohada hospitals in Tehran, Iran. Based on the pilot study and the sample size in similar studies, with a confidence level of 95% and a test power of 90%, a total of 60 individuals were enrolled. The subjects were randomly allocated into two groups of 30 each: The experimental and control groups. 

The inclusion criteria comprised an age range of 35 to 65 years, no substance abuse within the past 6 weeks, ASA I-II, lower limb orthopedic surgery under spinal anesthesia, absence of background diseases (e.g., ischemic heart disease, diabetes mellitus, and hypertension), absence of substance addiction according to the definition, normal body temperature, no history of tremors, seizures, tremors, or Parkinson's disease, no non-steroidal anti-inflammatory drug (NSAID) consumption 24 hours prior to surgery, absence of infections or wounds, absence of known allergies, no pregnancy or lactation, maximum surgery duration of 3 hours, and patient satisfaction. The exclusion criteria included the occurrence of adverse events requiring intervention within 24 hours after the surgery, requiring blood transfusion during surgery and anesthesia, and a decrease in the patient's core body temperature below 32 degrees Celsius during surgery. The ethical criteria for this study were adhered to by obtaining approval and consent from the Ethics Committee of Shahid Beheshti University of Medical Sciences, Tehran, Iran, with ethics code IR.SBMU.RETECH.REC.1401.754.

### 3.1. Procedure

After identifying suitable patients, necessary explanations regarding the procedure, benefits, and potential risks of participating in the study were provided, and written informed consent was obtained from the patients. The patients who met the necessary criteria were randomly assigned into two groups: The experimental and control groups, with 30 individuals in each group, while age and gender homogeneity were ensured. After patients entered the operating room, they were examined by an anesthesiologist, and their ASA class was determined. 

Following the placement of an intravenous (IV) line and standard monitoring, the skin was disinfected using an antiseptic solution. A transverse view with a probe covered by a sterile drape was obtained, and the brachial plexus network was identified between the anterior and middle scalene muscles. A 22-gauge needle was used under ultrasound guidance for the supraclavicular, infraclavicular, and axillary regions of the upper limb. In the control group, a mixture of 2 cc of 0.5% bupivacaine (20 milligrams) and 2 cc of sterile water was injected between the C5 and C6 cervical roots. In the experimental group, 2 cc of 0.5% bupivacaine and 2 cc of the perineural dexamethasone compound were injected. 

The success of motor block is defined as the inability to abduct the shoulder and sensory block with a pinprick test at the surgical site. The study was considered double-blind in the sense that, firstly, the patients and the anesthesiologist were unaware of which group each person was in, and only the researcher was aware that he/she was not in direct contact with the patients. Secondly, due to the doctor's lack of knowledge of injecting the medicine, it was pulled into the syringe by another person and handed over to the anesthesiologist. 

Pain intensity after surgery was assessed using the Visual Analog Scale (VAS) at 12 hours and 24 hours after the surgery. The VAS pain scale indicates the overall pain of patients. It is represented as a 10-centimeter line, and pain intensity is graded between 0 to 10 centimeters. A score of 0 indicates no pain, scores 1 to 3 indicate mild pain, scores 4 to 6 indicate moderate pain, and scores 7 to 10 indicate severe pain (8). The internal reliability of this tool has been reported as 0.85 to 0.95 (9). For data analysis, means, standard deviations, frequencies, tables, and charts were used to categorize and summarize the collected data. To assess the normal distribution of the data, the Kolmogorov-Smirnov test was employed due to the number of observations in each distribution. Considering the fulfillment of statistical assumptions, the independent *t*-test and repeated measures analysis of variance (ANOVA) were applied at a 95% confidence level using SPSS software version 22.

## 4. Results

The results of the Kolmogorov-Smirnov test indicated that the data distribution was normal (P > 0.05). The *t*-test results showed that there was no significant difference between the two groups regarding the VAS before the intervention (*t* = 0.97, P > 0.05). A two-way ANOVA (time × group) 3 × 2 was used for data analysis. The results are presented in Table 1. The findings revealed a significant main effect of group (F_1,58_ = 52.767, P < 0.001), a significant main effect of time (F_2,58_ = 3723.079, P < 0.001), and a significant interaction effect between group and time (F_2,58_ = 15.077, P < 0.001). The significant group effect indicates that there was a significant difference between the two groups regarding the VAS. 

**Table 1.** Results of Two-Way Analysis of Variance for the Visual Analog Scale in Two Groups

Variables	Degree of Freedom	Mean Square	F	P
**Group**	1	20.672	52.767	0.0001
**Time**	2	1613.333	3723.079	0.001
**Group × time**	2	6.533	15.077	0.001
**Error**	58	0.433		

**Figure 1.**
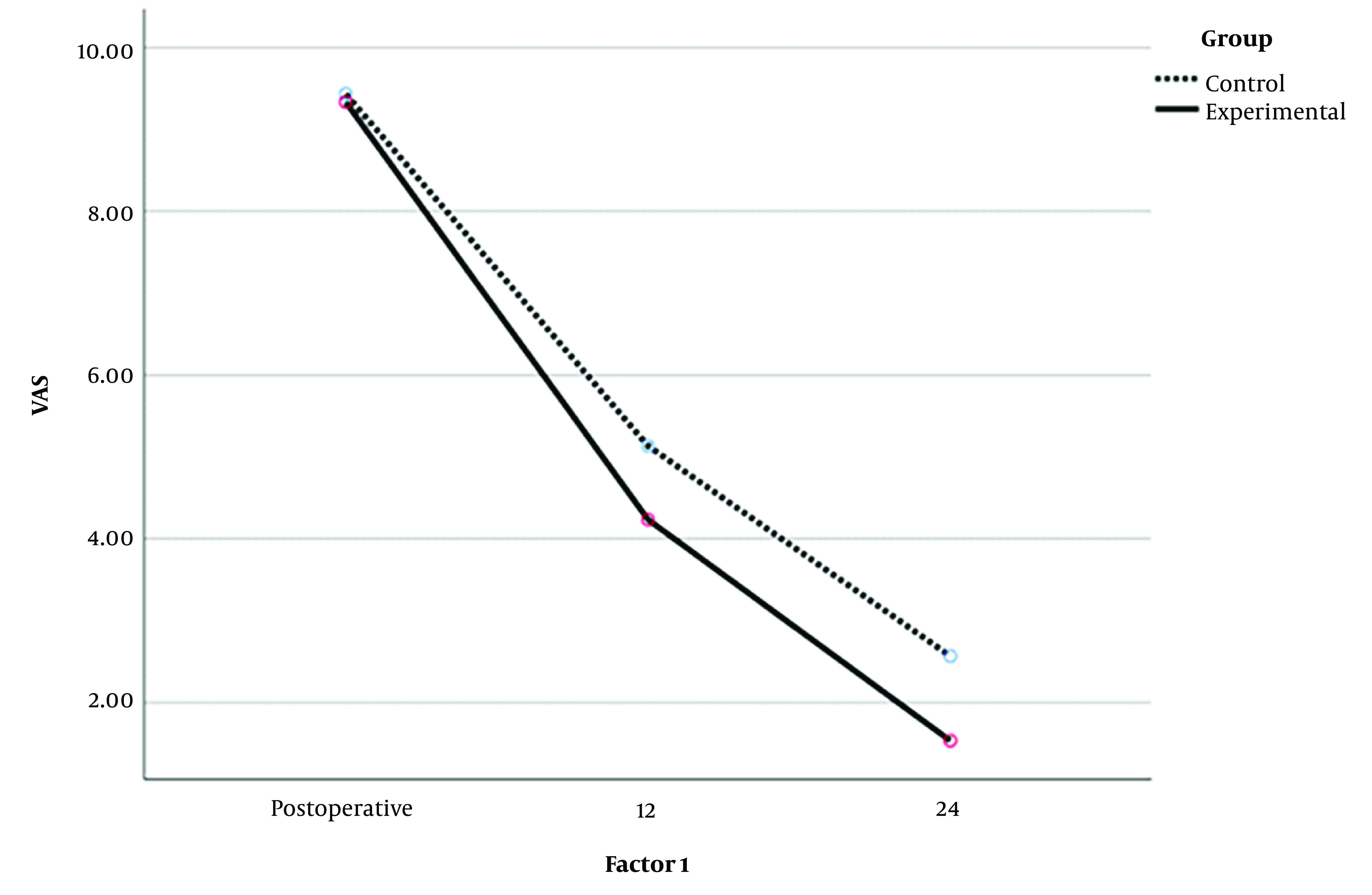
The Visual Analog Scale (VAS) of the two groups in the hours after surgery

Based on Figure 1, it can be stated that the experimental group experienced a greater reduction of pain than the control group. The significant time effect signified that the trend of pain reduction continued significantly in the experimental group at 12 and 24 hours after surgery (Figure 1). 

## 5. Discussion

The current study aimed to investigate the analgesic effects of the perineural dexamethasone compound in interscalene brachial plexus block after shoulder surgery. The results showed that the intensity of pain in the experimental group (dexamethasone) was lower than in the control group at 12 and 24 hours after surgery, indicating that dexamethasone was able to significantly reduce pain in patients. These findings are consistent with the results of studies by Pehora et al. (7), Yayik et al. (10), Badran et al. (11), and Kim et al. (12). For instance, Badran et al. demonstrated that adding 8 mg of perineural dexamethasone to 30 cc of bupivacaine 0.5% improved postoperative pain relief in shoulder surgery without apparent side effects and prolonged the duration of the block (11). They also suggested using bupivacaine with dexamethasone instead of liposomal bupivacaine (12). As an explanation of these findings, it can be stated that dexamethasone reduces pain by inhibiting the release of inflammatory mediators, such as interleukins and cytokines (13, 14). In other words, dexamethasone's systemic circulation and anti-inflammatory effects lead to a prolonged block that lasts up to 24 hours after surgery (15).

Furthermore, dexamethasone modulates peripheral pain threshold by increasing plasma beta-endorphin concentrations. This finding leads to a change in the level and release of beta-endorphins from the pituitary gland, thereby contributing to central pain control (16). Another study showed that dexamethasone could modify somatic pain and pain thresholds in patients with coronary artery disease (17). Additionally, the systemic administration of a potent corticosteroid, such as dexamethasone, can modify pain-relieving effects induced by selective mu-opioid receptor agonists. This finding suggests an important interaction between corticosteroids and the opioid system in at least the receptor level in the brain, and intrathecal administration of dexamethasone might modulate morphine's pain-relieving effects. This finding confirms that these effects might occur through the interaction or antagonism of dexamethasone with opioid receptors in a central area (18). Overall, it can be concluded that dexamethasone plays a role in reducing pain through its effects on various neural mechanisms in the spinal cord, peripheral nerve endings, brainstem-thalamus, hypothalamus, and brain cortex (16). 

Moreover, the findings of the present study are in contrast to the results of studies by Alboeh et al. (19), Mathiesen et al. (20), Cardoso et al. (21), and Lovich-Sapola et al. (1).

### 5.1. Conclusions

The discrepancy with previous research results can be attributed to differences in administration methods, dosage, and formulation of the drug. In general, it can be inferred that injecting 2 cc of dexamethasone can lead to pain reduction in shoulder surgery candidates in the long term, up to 24 hours after surgery. Therefore, this drug can be utilized as an effective pain-reducing medication in these patients.

## Clinical Trial Registration Code:

IRCT20170423033615N3.
